# High impact of implementation on school-based smoking prevention: the X:IT study—a cluster-randomized smoking prevention trial

**DOI:** 10.1186/s13012-016-0490-7

**Published:** 2016-09-17

**Authors:** Lotus Sofie Bast, Pernille Due, Pernille Bendtsen, Lene Ringgard, Louise Wohllebe, Mogens Trab Damsgaard, Morten Grønbæk, Annette Kjær Ersbøll, Anette Andersen

**Affiliations:** 1 National Institute of Public Health, University of Southern Denmark, Øster Farimagsgade 5A, 1353 Copenhagen, Denmark; 2 Danish Cancer Society, Strandboulevarden 49, 2100 Copenhagen, Denmark

**Keywords:** Implementation, Fidelity, School intervention, Smoking prevention, Adolescents, Intervention effect

## Abstract

**Background:**

Implementation fidelity describes how well an intervention is implemented in the real-world setting. Assessing implementation fidelity is essential in the understanding of intervention results. In most studies, implementation fidelity is measured insufficiently, though, not taking into account the complexity of the concept nor the intervention.

The objective of the present study was to develop an overall quantitative measure of implementation fidelity, to examine the degree of implementation fidelity and the association of implementation and effect of a randomized school-based smoking prevention trial—the X:IT study.

**Methods:**

A cluster-randomized trial testing is a multi-component intervention to prevent smoking among adolescents in 94 Danish elementary schools (51 intervention, 43 control schools). Participants were grade 7 pupils (mean age 12.5 years). Data was collected by electronic questionnaires among pupils at baseline (*n* = 4161), the first follow-up (*n* = 3764), and the second follow-up (*n* = 3269) and among school coordinators at intervention schools at the first and second follow-up (50 and 39 coordinators).

Intervention: The intervention included three components: (1) smoke-free school grounds, (2) smoke-free curriculum, and (3) parental involvement, contracts, and dialogues. Implementation fidelity was assessed by four domains: adherence, dose, quality of delivery, and participant responsiveness. These were combined into an overall school-wise implementation index. The association of implementation and smoking was examined by logistic regression analyses.

**Results:**

One fourth of the schools was characterized as high implementers of the program (all three components) at both first (12 schools, 24.0 %) and second follow-up (11 schools, 28.2 %). Implementation fidelity was strongly associated with smoking at the first and second follow-up, e.g., the odds for smoking at schools with high implementation both years were OR = 0.44 (95 % CI 0.32 to 0.68).

**Conclusions:**

Using an overall measure based on several aspects of implementation fidelity, we showed a negative graded association between implementation and smoking. This study suggests that higher degrees of implementation will improve the effect of the X:IT intervention. Studying the association between implementation and effect is extremely important; only by doing so, we can distinguish the quality of the intervention from the success of the implementation.

**Trial registration:**

Current Controlled Trials ISRCTN77415416

## Background

During the past decades there has been an increasing focus on measuring implementation fidelity within health research, and it is now well recognized that how an intervention is implemented affects the outcome [1, 2]. Assessing implementation can help us understand the results of an intervention, and ideally, conclusions about the effect should not be drawn without considering implementation. If an intervention fails to achieve the expected outcomes, it may be due to either the intervention itself or to lack of implementation [1, 3–5]. If the implementation is not assessed, the intervention may be determined as ineffective, when in reality, the lack of effect was due to implementation failure. Even when the expected effect is obtained, without assessing how it was implemented, one cannot be sure that the effect was due to the intervention itself [1, 3–5]. In order to gain a comprehensive picture of the implementation of a particular intervention, it is recommended to measure both structural aspects, such as adherence to the intervention and dosage of implementation, as well as procedural aspects, such as quality of delivery and participant responsiveness [4–6]. According to Dane and Schneider [4], adherence is the extent to which particular activities are implemented in accordance with how the program was designed. Dose is defined as the amount of program content received by participants, i.e., number of lessons received in a school-based intervention. Quality of delivery is how the program content is delivered; it is not directly related to prescribed content and delivery strategies, but rather to aspects such as teachers’ enthusiasm, preparedness, and attitudes towards the program. The assumption is that teachers who are prepared and positive towards the program do a better job implementing the program. Participant responsiveness is the extent to which participants are engaged by and involved in the content of the program, and program differentiation is an expression of the program uniqueness and is the extent to which the program theory can be distinguished from other programs [4, 6].

Several frameworks have been proposed to explain how these factors influence each other, and at the end—how it affects intervention outcome [5, 7–10]. There is no overall agreement, though, and just as not measuring implementation fidelity can lead to misunderstandings of the intervention effect, only measuring one aspect of implementation will leave the question whether this is sufficient to analyze associations between implementation and effect meaningfully [7].

Smoking is a major public health concern, and a large number of smoke preventive initiatives have been carried out. Most adult smokers initiated smoking in adolescence, and adolescent smoking strongly tracks into adulthood [11, 12]. In Denmark, adolescent smoking prevalence is still high; in 2014, 18 % of the 15-year-olds smoked regularly [13].

The school offers an ideal setting for preventive initiatives among children and adolescents [14], and over the past three decades, a large number of school-based smoking prevention programs have been launched internationally [11, 14, 15]. In Denmark, three large scale school-based smoking prevention interventions have been conducted and evaluated scientifically, unfortunately none of them with positive results [16–18]. Internationally, the intervention effects have been inconsistent [11, 14, 15], which may partly be explained by lack of implementation [19], although comprehensive studies of the implementation of school-based interventions have been rare [4, 6, 20]. Previous studies lacked consensus on how to measure implementation, and many studies reported on only one or two dimensions of implementation, most often adherence or dose, which is insufficient to give a comprehensive picture of the implementation [21, 22]. For example, measuring number of lessons taught in a school-based program does not provide information on how teachers delivered the lessons or whether pupils felt engaged by the curriculum. In 2013, Harn et al. examined the current state of knowledge on implementation fidelity in school-based prevention and concluded that although it has become more and more common to report implementation fidelity, still only half of the examined studies reported some kind of implementation measure [23].

A lot of the implementation of school-based prevention activities often relies on the teachers as implementers, along with the many other tasks of teaching [9, 24], and at the same time, the demands on schools are rising [25–27]. Therefore, implementation of a new initiative in an already challenged school setting may be difficult, and the degree of implementation of school-based prevention varies greatly [6, 28, 29].

The literature on implementation of smoking prevention initiatives is sparse. In 2008, Sy and Glanz’s process evaluated the SPLASH Project and found that 71.4 % of the teachers implemented the program with high fidelity. Here, implementation fidelity was defined as dosage of delivery [30]. In another trial—ASSIST—implementation fidelity was reported by aspects of adherence to the intervention protocol, quality of delivery, participant responsiveness, and variable delivery across sites. The overall implementation was high, although there were variations between schools [31]. More recently, Trigwell et al. [32] evaluated the implementation fidelity of SmokeFree Sports through aspects of reach, dose of delivery fidelity (whether the intervention was delivered as intended), acceptability, and sustainability. Each aspect of implementation was reported, and a total score for each program session was calculated as percentages for comparison across components. The implementation fidelity measure was categorized into low (0–33 %), medium (34–66 %), and high (67–100 %) implementation, and the overall implementation fidelity score was 57.8 %, while 28 % of the sessions was scored with high fidelity [32].

The most comprehensive evaluation of implementation fidelity and smoking prevention was from the Tobacco Prevention and Education Program in Oregon [33]. Here, implementation fidelity was defined as the presence of six predefined implementation criteria: tobacco-free school policies, family involvement, community involvement, tobacco prevention curriculum instruction, teacher/staff training, and pupil tobacco use cessation support. Measures of each of the six implementation criteria were summed into a composite implementation score. Based on natural cut points within the distribution of data, the schools were divided into three implementation groups, and significantly greater declines in smoking prevalence among year 8 pupils were found in schools that rated medium or high [33].

We have been unable to find studies in the area of school-based smoking prevention, which use a comprehensive and theoretically based combined measure of implementation, covering several intervention components, i.e., adherence, dose, quality of delivery, and participant responsiveness. Most importantly, there is a lack of studies investigating the importance of implementation fidelity on smoking behavior.

In 2010, the Danish Cancer Association launched a large smoking prevention intervention for 13- to 15-year-olds—the X:IT study. It is a school-based multi-component program to prevent smoking among adolescents. The effect of the program was evaluated using a cluster-randomized controlled trial involving schools from all over Denmark. Intention to treat analyses have shown an overall significant effect of the X:IT intervention [34], but we do not know whether the effect was differential according to implementation fidelity. In order to investigate whether the full potential of the intervention had been reached, it is therefore important to study implementation of the intervention.

The purpose of the present study was to develop an overall quantitative measure of implementation fidelity, to examine the degree of implementation fidelity and the association of implementation fidelity and effect of the X:IT intervention.

## Methods

**Fig. 1:**
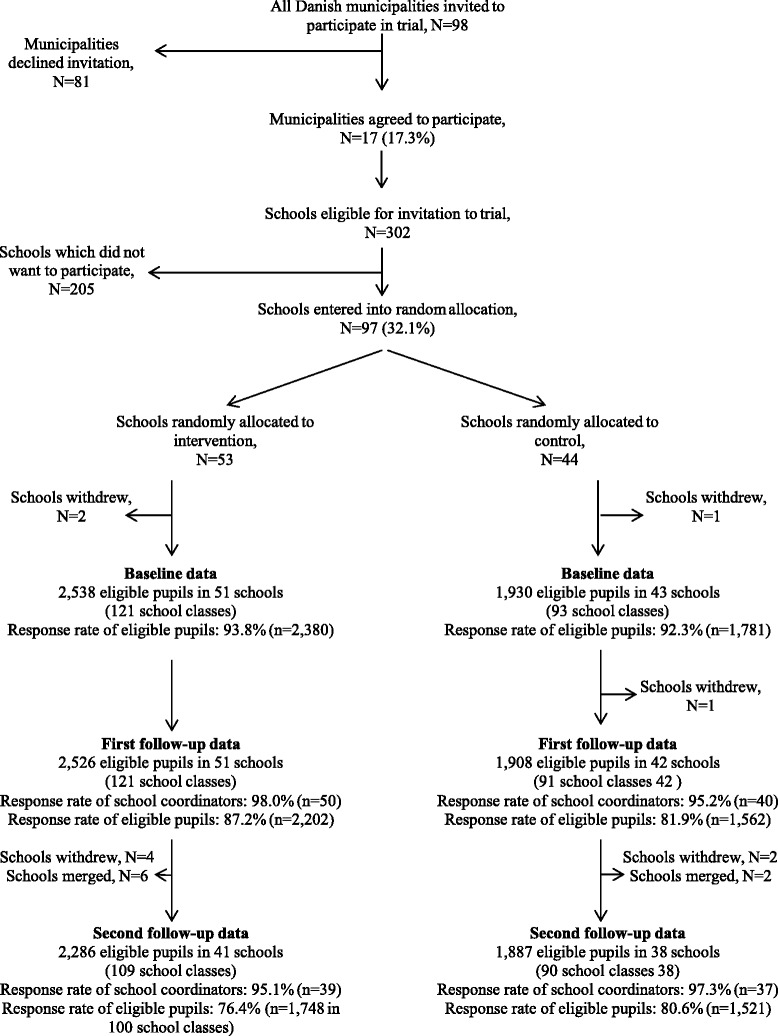
Flow diagram of the recruitment, randomization, and participation of municipalities, schools, and pupils in the X:IT study

The X:IT study was a large randomized trial with three successive data collections. All municipalities in Denmark were invited to participate in the X:IT study (*N* = 98), and 17 of them agreed. Within these municipalities, 302 eligible schools were invited and 97 joined the study (32.1 %). The randomization was stratified by municipality and performed by drawing lots. Three schools withdrew after randomization leaving 51 intervention and 43 control schools. All grade 7 pupils (mean age 12.5 years) were invited to participate. Data were collected by electronic questionnaires among pupils and coordinators at schools. We followed pupils over time by information of their name, birthday, class, and school. Pupils were informed that completion of questionnaires was optional and that any information they provided would be anonymous and treated with confidence. The process of recruitment, randomization, and data collections is shown in Fig. 1. The response rates are based on number of responses out of number of eligible pupils at baseline and the first and second follow-up.

Baseline data were collected at the beginning of grade 7 (fall 2010), the first follow-up at the end of grade 7 (spring 2011), and the second follow-up at the end of grade 8 (spring 2012). At baseline, there were 2538 eligible pupils in the 51 intervention schools (response rate = 93.8 %) and 1930 in the 43 control schools (response rate = 92.3 %).

At the first follow-up, 2526 pupils were eligible from the 51 intervention schools (response rate = 87.2 %). One control school dropped out between baseline and the first follow-up, leaving 42 schools with 1908 pupils eligible (response rate = 81.9 %). Further, we used data from the school project coordinators—preferably a teacher—from each intervention school (responses 50 of 51 schools).

Between the first and second follow-up, four intervention schools dropped out of the study. Further, due to a nationwide political reform leading to merging of schools, the number of schools in the intervention group declined. At the second follow-up, 2286 pupils eligible for study led to 1748 responses (76.5 %) from the 41 intervention schools and 1887 pupils eligible at 38 control schools, giving 1521 responses (80.6 %). The school project coordinator surveys were answered by 39 of 41 coordinators at the second follow-up.

For the analyses of implementation fidelity, we used the first and second follow-up data from intervention schools. Data from control schools were included in the effect analyses.

### Measurement of implementation

In the present study, implementation fidelity was defined as the degree to which the program was implemented as intended and assessed by the following: adherence to the intervention, dose, quality of delivery, and participant responsiveness [6]. Adherence refers to the extent to which core intervention components are delivered in accordance with the program. Dose is the amount of the intervention components received by participants. Quality of delivery refers to how the providers delivered the program components. Participant responsiveness reflects the extent to which participants were engaged by and involved in the activities of the program [6].

X:IT consists of three main intervention components: (1) smoke-free school grounds, (2) smoke-free curriculum, and (3) parental involvement comprising smoke-free contracts and dialogues [35].

#### Smoke-free school grounds

Schools had to be smoke-free for pupils and teachers both indoor and outdoor on school grounds, and schools had to plan enforcement strategies to control pupils smoking at school grounds.

We measured the following: adherence to this component by assessing whether it was allowed for pupils and teachers to smoke during school hours; dose as the amount of smoking experienced at school by measures of how often pupils saw other pupils or teachers smoke; and quality of delivery as enforcement of smoking rules and participant responsiveness by pupils’ attitudes to smoking rules (Table 1).

**Table 1:** Overview of implementation fidelity measures and data sources of the X:IT study by program components: smoke-free school grounds, curriculum, and parental involvement

Fidelity measures	Adherence	Dose	Quality of delivery	Participant responsiveness
Intervention component				
Smoke-free school grounds				
Definition	Rules for smoking at school	Exposure to smoking at school	Enforcement	Attitudes to smoking rules
Measures (response categories)	Are pupils allowed to smoke during school hours? (*Yes + yes outside school grounds vs. no*) Are teachers allowed to smoke during school hours? (*Yes vs. yes if invisible to pupils vs. no*)	How often do you see pupils smoke different places at school? (*Daily + sometimes vs. never*) How often do you see teachers smoke different places at school? (*Daily + sometimes vs. never*)	How often do teachers control pupil smoking inside the school, outside on school grounds, and outside the school grounds? (*Daily + weekly + monthly vs. less + never*)	State your opinion; Pupils/teachers should be allowed to smoke during school hours? (*Totally agree + agree vs. neither vs. disagree + totally disagree*)
Data source	School coordinator questionnaire	Pupil questionnaire	School coordinator questionnaires	Pupil questionnaires
Smoke-free curriculum				
Definition	Eight mandatory lessons on smoking related issues delivered	Number of lessons on smoking related issues received	Quality assessment of “Up in Smoke” (curriculum material)	Attitudes to teaching about smoking related issues
Measures (response categories)	For each school class, how many of these lessons did the class have? (*Mandatory eight lessons + more vs. less + none*)	How many hours of teaching did you have? (*None + 1–3 h + 4–6 h vs. 7–9 h + 10 h or more*)	How well did the ‘Up in Smoke’-material work? (*Very well + well vs. parts of it not so well + not well + not well at all vs. did not teach/use the material*)	How well did you like the teaching? (*Very much + okay vs. did not like + did not like at all vs. no teaching*)
Data source	School coordinator questionnaire	Pupil questionnaire	School coordinator questionnaire	Pupil questionnaire
Parental involvement				
Definition	a) Smoke-free contracts b) Smoke-free dialogues c) X:IT presented at a parents meeting		Parental involvement in a) The contract and b) The smoke-free dialogue	
Measures (response categories)	a) Smoke-free contract (*Fulfilled and signed vs. not fulfilled and signed*) b) Did you have a smoke-free dialogue with your parents? (*Yes vs. no + do not see/have parents*) c) For each school class, was X:IT presented at a parents’ meeting? (*Yes vs. no*)		a) Were your parents positive towards the smoke-free contracts? (*Yes + yes partly vs. no + do not know*) b) Did your parents ever: - State their opinion on smoking? - Listen to your opinion? - Ask if you have tried smoking? - Encourage you not to smoke? - Ask if you have been offered cigarettes? - Or encourage you to talk about smoking if needed? (*Yes highly + yes vs. no*)	
Data source	Copy of contract, pupil and school coordinator questionnaires		Pupil questionnaire	

#### Smoke-free curriculum

The educational material “Up in Smoke” (www.op-i-roeg.dk) was based on self-efficacy training and preparation of outcome expectancies and designed to be used in subjects such as science, humanities, and social sciences. The program included eight teaching lessons a year with detailed study guidelines for each educational year. Adherence was reported by coordinators stating whether each school class had had the mandatory lessons; dose as proportion of classes where at least half of pupils remembered having had at least seven lessons; quality of delivery of the curriculum: coordinators should state how well they thought the curriculum worked, and we asked pupils for their responsiveness to the teaching by stating how well they liked it.

#### Parental involvement

The parental involvement component comprised smoke-free contracts between pupils and an adult person—preferably a parent—and smoke-free dialogues between pupils and their parents. By signing the contract, the pupil promised to stay smoke-free for the following year. All pupils, who remained smoke-free, participated in a lottery to win a prize. Most often the prize was an iPod, iPad, or a gift certificate. All pupils had an equal chance of winning the prize, which was provided by the municipalities. Further, teachers presented the X:IT study at parent meetings at the beginning of each school year. We assessed adherence by number of returned contracts and pupils responses as to whether they had signed a contract and by coordinator reports on whether the X:IT study was presented at a parent meeting at the school. Quality of delivery was assessed by pupils’ answers to whether parents were positive towards the contracts. This was measured by six items concerning different aspects of the smoke-free dialogue and combined to one single count measure on whether the pupil had answered *very much or to some degree* to at least four out of the six items. In example, “did your parents ever ask if you have tried smoking or encouraged you not to smoke” (Table 1). Dose and participant responsiveness for the parental involvement component were not included in the quantitative data but explored qualitatively and thus not a part of the implementation measure.

### Implementation index

We constructed an implementation index differentiating intervention schools into categories of high, medium, or low implementation fidelity. We combined items within each of the three program components, separately for pupils and school coordinators, and across all four implementation concepts (adherence, dose, quality of delivery, and participant responsiveness). All items described in Table 1 were included, first aggregated at the school level, then rescaled ranging from zero to one, and summed into indices. There were no weighting of items, so all items contributed equally to the indices.

For each program component, there were two indices: one based on school coordinator responses and one based on pupils’ responses. The applicability of this approach was tested trough confirmatory factor analyses [36]. The root mean square error of approximation (RMSEA) was 0.072. Ideally, for a good model fit, it should be below 0.05, but some researchers argue that values up to 0.08 are acceptable [37]. The standardized root mean square residual (RMSR) was 0.055, where a value of 0.08 or less being indicative of an acceptable model [38]. The Bentler Comparative Fit Index (CFI) was 0.790, where a CFI value of 0.90 or larger is generally considered to indicate acceptable model fit [38].

The definition of whether a component was implemented or not was based on conceptual discussions, and cut points for implementation were decided before analyses. For example, implementation of the parental involvement component was measured among pupils by four items: (1) whether they had signed a smoke-free contract, (2) whether their parents were positive towards the contract, (3) whether they had had a smoke-free dialogue, and (4) whether their parents were positive towards the dialogue. We regarded this component as implemented if pupils had responded positive on at least three out of four items, thereby assuming that this would measure implementation (adherence and/or quality of delivery) of a contract as well as a dialogue.

In the overall implementation index, a component was regarded as implemented only if pupils as well as school coordinators had responded positively on the implementation of that particular component.

#### Smoke-free school grounds

Three items were included for pupils: how often do you see pupils smoke at school; how often do you see teachers smoke at school; and attitudes to smoking rules stating whether or not pupils/teachers should be allowed to smoke during school hours. School coordinators answered to the following three aspects: are pupils allowed to smoke during school hours; are teachers allowed to smoke during school hours; how often do teachers control pupils smoking. This resulted in two indices for smoke-free school grounds (pupils and teachers), which ranged from zero to three, and was regarded as implemented at a cut point of 1.5.

#### Smoke-free curriculum

Implementation of the curriculum was measured by two items for pupils: how many hours of teaching did you have and how well did you like the teaching. Coordinators answered two items: whether the mandatory eight lessons were delivered and how well the curricular material worked. The curriculum was regarded as implemented if pupils and coordinators answered positively to at least one of the items, and therefore, the cut point was set at 1.

#### Parental involvement

For pupils, four items measured parental involvement: whether or not parents (or another adult) had signed the smoke-free contract; whether or not the smoke-free dialogue was held; and their parents’ attitude to the contract and the dialogue. Parental involvement was defined as implemented if they had answered “yes” to three of the four items. Hence, the cut point was set at 3.0. The other part of parental involvement was the school coordinators’ reporting on presentation of X:IT at parent meetings, which was implemented if the coordinator had answered “yes,” with a cut point of 1.0. Based on all the abovementioned items from pupils and school coordinators, we combined indices into an overall implementation index for each school. An intervention component was implemented only if both school coordinators and pupils said that it was. Schools in the high implementation group had implemented all of the three components according to the demands of the X:IT study; schools in the medium implementation group had implemented two out of three components; and low implementation schools had implemented only one or none of the components as required.

### Outcome measure

The outcome measure was “current smoking” based on the question: “How often do you smoke?” at the first and second follow-up. We dichotomized answers into daily + weekly + monthly + less frequent vs. never.

### Data analyses

The outcome measure was a summarized binomial response variable derived by aggregating current smoking at school level. An observation in the data set represented a school with number of current smokers and number of participating pupils in the school [39].

Effect of implementation fidelity on smoking was examined through logistic regression analyses with number of current smokers at school level as number of events and number of pupils participating at school level as the number of trials. To explore differences in implementation fidelity between schools, the implementation index was constructed at school level. The determinant variable was implementation fidelity, which differentiated the schools into the following: high, medium, or low implementation, and the control group was used as reference. At the second follow-up, we took into account the implementation fidelity of year 1 by combining implementation status of both first and second year; we joined medium and low implementation schools into the same group and build a combined variable of the first and second year implementation: schools with (1) high implementation both years, (2) high implementation at one follow-up point, and (3) medium or low implementation at both follow-ups.

Cut points for the implementation index were checked by changing the cut points for all six indices to half of the possible value, which eased the criteria for implementation of the parental component, and by changing the cut point for all indices to two thirds of the possible value, which meant a tightening of the demands for the smoke-free school grounds and the curricular activities.

All analyses were performed with SAS 9.3 software, using the PROC CALIS procedures for factor analyses and PROC GENMOD for logistic regression analyses.

## Results

### Smoke-free school grounds

According to school coordinators, the implementation of the smoke-free school component increased over the study period: After 2 years of study, two thirds (66.7 %) of the intervention schools had implemented the total smoking ban for pupils compared to 59.0 % the first year (Table 2). At the majority of schools, teachers were allowed to smoke if invisible to pupils; 76.0 % at the first- and 66.7 % at the second follow-up. The proportion of schools with a total smoking prohibition for teachers rose from 16.0 to 25.0 %. According to school coordinator responses, teachers controlled pupils smoking outside on school grounds every day or weekly at 77.0 % of the schools at the first follow-up and at 86.5 % of schools a year later. At the first follow-up, more than half of the pupils reported seeing other pupils (60.9 %) and teachers (42.8 %) smoking outside at school grounds. There were no changes to the second follow-up.

**Table 2:** Implementation of the X:IT intervention at the first and second follow-up by program components: smoke-free school grounds, curriculum, and parental involvement

Fidelity measures	Adherence			Dose			Quality of delivery			Participant responsiveness
Intervention component										
Smoke-free school grounds										
Answered by pupils				See pupils smoke daily or sometimes						Pupils should be allowed to smoke at school grounds
				Inside at school	Outside at school grounds	Outside school grounds				
First follow-up (2202 pupils)				237 pupils (11.6 %)	1253 pupils (60.9 %)	1558 pupils (75.1 %)				261 pupils (13.2 %)
Second follow-up (1748 pupils)				287 pupils (17.4 %)	953 pupils (57.2 %)	1242 pupils (74.5 %)				230 pupils (15.9 %)
				See teachers smoke daily or sometimes						Teachers should be allowed to smoke at school grounds
				Inside at school	Outside at school grounds	Outside school grounds				
First follow-up				601 pupils (29.4 %)	896 pupils (42.8 %)	998 pupils (48.3 %)				286 pupils (14.5 %)
Second follow-up				428 pupils (25.1 %)	731 pupils (43.6 %)	915 pupils (54.7 %)				237 pupils (16.5 %)
Answered by school coordinators	Total smoking prohibition pupils						Teachers control pupils smoking daily or often			
First follow-up (50 schools)	29 schools (59.0 %)									
Second follow-up (39 schools)	24 schools (66.7 %)									
	Total prohibition teachers	Allowed if invisible	Allowed				Inside at school	Outside at school	Outside school grounds	
First follow-up	8 schools (16.0 %)	38 schools (76.0 %)	4 schools (8.0 %)				36 schools (75.0 %)	37 schools (77.0 %)	24 schools (50.0 %)	
Second follow-up	9 schools (25.0 %)	24 schools (66.7 %)	3 schools (8.3 %)				30 schools (81.1 %)	32 schools (86.5 %)	23 schools (62.2 %)	
Smoke-free curriculum										
Answered by pupils				Number of classes where at least half of pupils remember having 7 to 9 or more lessons						Liked the teaching
First follow-up (2202 pupils in 121 classes)				26 classes (21.5 %)						1794 pupils (82.3 %)
Second follow-up (1748 pupils in 100 classes)				8 classes (8.0 %)						1340 pupils (77.6 %)
Answered by school coordinators	Mandatory 8 lessons or more						Material worked well			
First follow-up (50 schools, 121 classes)	93 classes (80.2 %)						28 schools (60.9 %)			
Second follow-up (39 schools, 100 classes)	58 classes (65.9 %)						17 schools (47.2 %)			
Parental involvement										
Answered by pupils	Smoke-free contract	Smoke-free dialogue					Parents positive towards smoke-free contracts	Parents expressed their opinion on the smoke free dialogue		
First follow-up (2202 pupils in 121 classes)	1818 pupils (83.2 %)	1652 pupils (79.0 %)					1442 pupils (68.7 %)	1437 pupils (70.5 %)		
Second follow-up (1748 pupils in 100 classes)	1249 pupils (73.9 %)	1300 pupils (78.7 %)					1126 pupils (66.7 %)	1129 pupils (70.7 %)		
Answered by school coordinators	X:IT presented for parents									
First follow-up (121 classes)	111classes (94.9 %)									
Second follow-up (100 classes)	84 classes (88.4 %)									

Numbers are percentages of item responses

### Smoke-free curriculum

The implementation fidelity of the smoke-free curriculum was 80.2 % the first year and 65.9 % the second year according to coordinator responses. At both follow-ups, pupils remembered much smaller numbers of curricular activities than the coordinators. About half of the coordinators (60.9 % at the first follow-up and 47.2 % at the second follow-up) reported that the material worked well, while larger proportions of pupils liked the lessons based on “Up in Smoke” (82.3 % at the first year and 77.6 % at the second year).

### Parental involvement

Largely, the parental component was well implemented and fairly stable over time. At the first follow-up, 83.2 % of pupils (*n* = 1818) signed a smoke-free contract and 79.0 % (*n* = 1652) said that they had had a smoke-free dialogue with their parents. At the second follow-up, it was 73.9 % (contracts) and 78.7 % (dialogues). The study was presented at parent meetings in most school classes: 94.9 % the first year and 88.4 % the second year.

### School-level implementation

About one fourth of the schools were characterized as high implementers (all three components) at both first (24.0 %) and second follow-up (28.2 %). The proportion of schools in the low implementation group was considerably higher at the second follow-up (48.7 %) compared to the first follow-up (32.0 %).

In the medium implementation group at the first follow-up, most of the schools had not implemented the parental involvement component sufficiently (10 out of 22), while 6 schools had not implemented the smoke-free curriculum and 5 schools did not implement the smoke-free environment (data not shown). The patterns were similar at the second follow-up.

### Effect of implementation on smoking

There was a graded and significant negative association between implementation and smoking at the first follow-up (*p* = 0.012) and in analyses combining results from the first and second follow-up (*p* = 0.035).

**Fig. 2:**
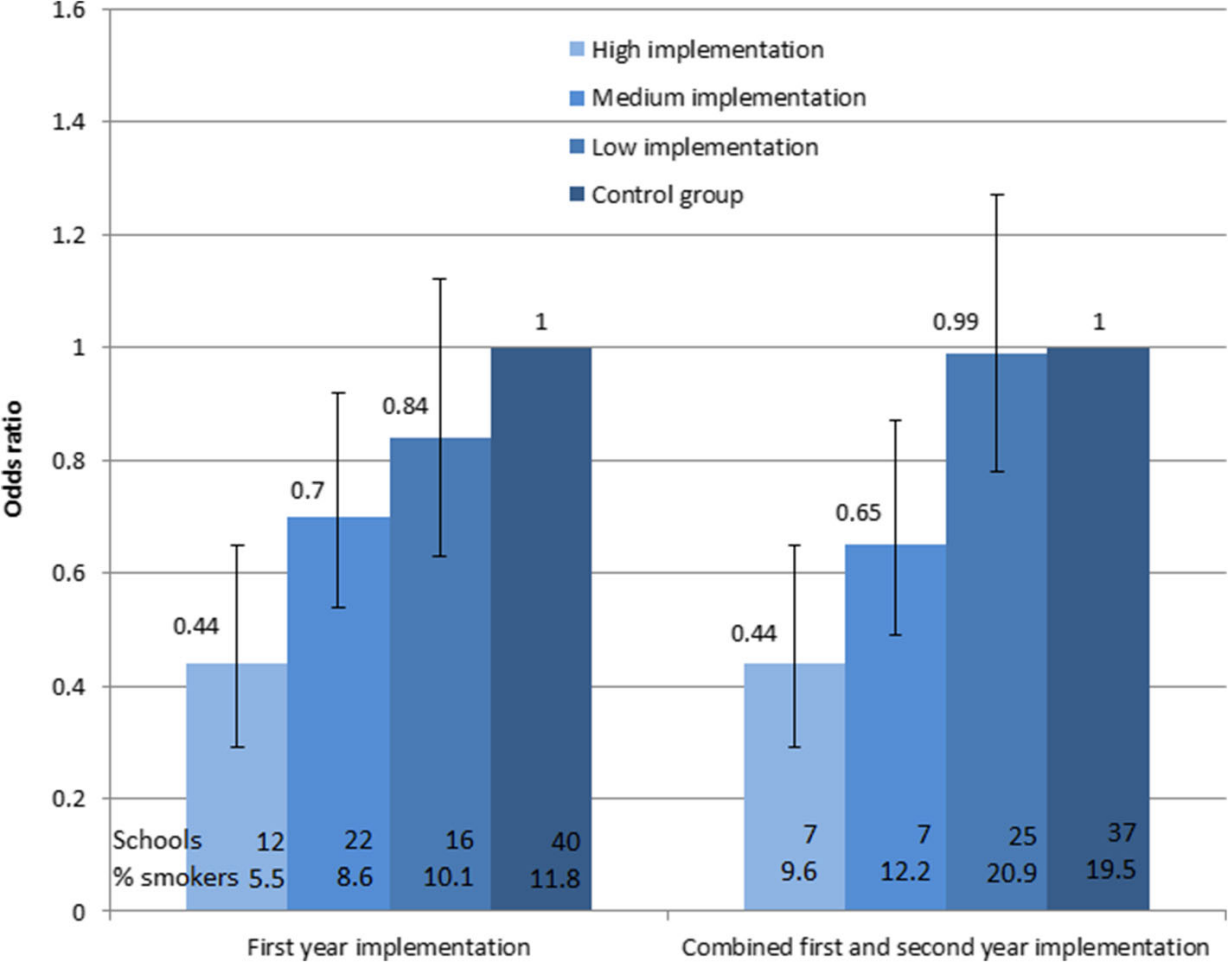
Smoking status at school level after 1 and 2 years of implementation of the X:IT program in relation to implementation status (high, medium, and low). Odds ratios (OR) and 95 % confidence interval

At the first follow-up, the OR for smoking at schools with high implementation was 0.44 (95 % CI 0.29 to 0.65, *p* < 0.001) compared to control schools (Fig. 2), while OR for smoking at schools with medium implementation was 0.70 (95 % CI 0.54 to 0.92, *p* = 0.012). The difference between high and medium implementation was tested significant with a *p* value of 0.031. At schools with low implementation, there was no significant association to smoking status, OR = 0.84 (95 % CI 0.63 to 1.12, *p* = 0.236).

High implementation both years was strongly associated to smoking; OR = 0.44 (95 % CI 0.32 to 0.68, *p* < 0.001) (Fig. 2), while medium implementation (high implementation 1 year in combination with a year of medium or low implementation) showed a less strong association; OR = 0.65 (95 % CI 0.43 to 0.97, *p* = 0.035), although not statistically different from the high implementation (*p* = 0.173).

Sensitivity analyses using different cut points for the implementation index showed results in the same directions.

## Discussion

This study found that it was possible to develop and use an overall quantitative measure of implementation built on existing implementation frameworks within implementation science [4, 6]. Implementation of the main program components in the X:IT study was measured by aspects of adherence, dose, quality of delivery, and participant responsiveness and varied greatly across components and over time. One fourth of the schools implemented the program with high fidelity. Schools, which implemented the intervention in close accordance with the aim and directions of the intervention program, were highly effective in preventing smoking among adolescents compared to control schools, and there was a graded negative association between implementation fidelity and smoking prevalence among adolescents.

According to school coordinators, the proportion of schools implementing the smoke-free school component increased over the study period. The smoke-free curriculum was implemented with high fidelity the first year but dropped markedly the second year; while parental involvement, based on contracts and dialogues, was implemented with high fidelity and stable during the intervention period.

The implementation of the smoke-free school component increased over time. Implementation of this component required the whole school to implement and enforce the strict anti-smoking rules for pupils as well as for teachers. Such a structural change at the school may require a longer implementation period, than a component delivered in the classroom setting, which requires action from a single teacher only [40]. Further, the implementation of this component may have been challenged by inclusion of teachers own smoking habits. Implementation of the other components of the X:IT intervention may have contributed to the change of attitudes towards smoke-free school grounds over the study period, which can be reflected in the increased implementation over time of this particular component.

In accordance with the literature [6, 41, 42], the implementation of the curricular activities was highest the first year and declined at year 2. This may partly be due to the fact that a lot of work was invested in the initiation of the project including kick-off training workshops for school coordinators. The parental involvement component was well implemented continuously over both years. This component was inspired by two Nordic studies: the Norwegian study “BE smokeFREE” [43] and the Swedish study “Tobacco Free Duo” [44]. In the Swedish study, the smoke-free contract component in itself seemed to reduce smoking prevalence by almost 50 %. None of these studies reported on implementation. As part of the X:IT intervention, pupils who remained smoke-free for 1 year were registered in a lottery to win a prize, which may partly explain the high implementation fidelity of the parental component.

In 2008, Durlak and DuPre reviewed more than 500 health promotion and prevention studies and found that usually, there is a large variability of implementation across program providers within the same study [1]. This was confirmed in the X:IT study, as we found large variations across schools.

The graded association between implementation and smoking prevention, which was found in this study, has previously been shown by Rhode et al. [33], although our effect sizes seemed stronger. This may be due to differences in the design of the intervention, as well as differences in measures. For example, no pupil reportings of implementation were included by Rhode et al. The X:IT intervention was developed years after the other intervention and based on elements proven effective by literature in the meantime. This strong and theoretical anchoring may partly lead to the larger effect sizes. To our knowledge, no other studies on school-based smoking prevention interventions have used a comprehensive and theoretically based combined measure of implementation fidelity, which covered all intervention components, and tested the association of implementation and smoking status. We found that schools with high implementation fidelity were very effective in preventing smoking uptake among adolescents compared to control schools. Our study indicates that using an overall implementation measure may be a feasible method when investigating the role of implementation on the effect of intervention studies addressing adolescent smoking.

### Limitations and strengths

X:IT is a large randomized trial across 94 intervention and control schools with more than 4500 pupils. The response rates were high, above 75 % at all data collection points. We carefully examined implementation across several domains of implementation and over time, thereby taking into account that implementation of an intervention needs to be comprehensive and that it is likely to change over time. As recommended by Dane and Schneider [4], we included aspects of adherence to the intervention, dose, quality of delivery, and participant responsiveness; the fifth aspect—program differentiation—was not included in our implementation index, due to the slightly different nature of the concept. Program differentiation can be seen as an analytical process before, during, and/or after the measurement of implementation, rather than a dimension of the implementation fidelity measure itself [7].

Self-reporting is regarded as a valuable method of gaining insight into pupils’ explicit attitudes, experiences, and behaviors. Studies of adolescent self-reported smoking habits have shown good validity against biochemical measures [45, 46]. To deal with the tendency to overestimate implementation in self-reports [47, 48], we used data from both coordinators (implementers) and pupils (participants) to create a combined measure of the implementation fidelity. Any discrepancies between the two sources of data were handled by regarding a main intervention component as implemented only if coordinators as well as pupils responded that it was implemented. This procedure may have resulted in underestimating the actual implementation fidelity. Implementation fidelity aspects of more structural character, i.e., dose, can relatively easily be conceptualized as number of completed tasks or number of lessons taught, whereas more process-related measures as quality of delivery or participant responsiveness are more difficult to measure [8]. For example, we conceptualized participant responsiveness of the curricular component as to whether the pupils liked the teaching that they had received. Although liking might not fully reflect the pupils’ involvement, it still to some degree reflects whether the pupils felt engaged with the curricular activities. More observational data collection methods could be preferable for collecting this kind of information, although not always feasible in large studies with multiple intervention sites [49].

All Danish municipalities were invited to the X:IT study, and among those municipalities who agreed to participate, all schools were invited. This procedure could have resulted in selection bias, but municipalities are large entities and non-attendance was mainly excused by lack of administrative time at the municipal level. Within municipalities, participating schools were randomly allocated to either intervention or control group. No pupils actively withdrew, and pupils not attending school the day of the survey were encouraged to answer the questionnaire another day.

Our measure of implementation is based on the work by Dusenbury and Dane and Schneider [4, 6]. They consider the elements of implementation (adherence, dose, quality of delivery, participant responsiveness, and program differentiation) as complementary and necessary for a comprehensive picture of the implementation of an intervention [4, 6]. Carroll et al. [50] proposed a conceptual framework for implementation adding concepts to the elements of Dusenbury and Dane and Schneider. According to Carroll et al., some of these elements could work as moderators for the others. For instance, implementation fidelity may be moderated by intervention complexity, quality of delivery, and participant responsiveness [50]. A limitation of our study is that we did not take into account the proposed relationships between the elements of implementation fidelity in the analyses. A further examination of how the different aspects of implementation fidelity influence each other is of great interest for the field of implementation research; unfortunately, this goes beyond the scope of this particular paper.

### Implications for research

By the use of one combined implementation index, we were able to identify implementation of a complex multi-component intervention at each school and to compare schools of different implementation fidelity. The applicability of this approach should be tested in other studies. It seems that the effect of the X:IT intervention was dependent on implementation of each of the main components: smoke-free school grounds, smoke-free curriculum, and parental involvement comprising smoke-free contracts and dialogues. The mechanisms behind and how the components work together should be explored in future research. Also, it should be examined which school settings provide the most optimal conditions for implementation of the intervention.

### Implications for practice

Several smoking prevention programs have been introduced to schools during the last decades, but many of them have not been effective. In Denmark, the X:IT study has been the first smoking preventive program which has shown significant effect on adolescent smoking [34]. Our study demonstrated that the effect of the intervention is strongly and positively associated with the degree to which the schools implement the program as intended by the program developers. The optimal effect of the X:IT intervention was found when all three main components was implemented as intended by program developers, i.e., when school grounds were totally smoke-free for both pupils and teachers; the mandatory 8 h of curricular activities was provided; and pupils had smoke-free dialogues and promised to stay smoke-free for the following year through the smoke-free contracts. It seemed that the intended mechanisms behind the X:IT intervention was well theorized, and the multi-component structure of the program was beneficial. Lack of implementation of one or more components reduced the effect substantially.

Implementation fidelity varied considerably between schools, showing that ensuring good implementation fidelity across multiple settings can be challenging. Learning from schools with high implementation fidelity may help us to improve the implementation of the X:IT intervention in the future.

## Conclusions

It was possible to develop an overall measure of implementation. The measure demonstrated a variation in implementation fidelity between schools and over time and revealed a graded negative association between implementation of the X:IT intervention and adolescent smoking. Any lack of effect of the X:IT intervention may therefore be ascribed to lack of implementation rather than to deficiency of the intervention.
